# Statistical Method to Detect Tuberculosis Outbreaks among Endemic Clusters in a Low-Incidence Setting

**DOI:** 10.3201/eid2403.171613

**Published:** 2018-03

**Authors:** Sandy P. Althomsons, Andrew N. Hill, Alexia V. Harrist, Anne Marie France, Krista M. Powell, James E. Posey, Lauren S. Cowan, Thomas R. Navin

**Affiliations:** Centers for Disease Control and Prevention, Atlanta, Georgia, USA

**Keywords:** tuberculosis and other mycobacteria, bacteria, respiratory infections, molecular epidemiology, genotype, endemic clusters, outbreaks, low-incidence setting, statistical method, United States

## Abstract

We previously reported use of genotype surveillance data to predict outbreaks among incident tuberculosis clusters. We propose a method to detect possible outbreaks among endemic tuberculosis clusters. We detected 15 possible outbreaks, of which 10 had epidemiologic data or whole-genome sequencing results. Eight outbreaks were corroborated.

We previously reported use of data from the National Tuberculosis Genotyping Service in the United States to predict outbreaks among incident clusters of tuberculosis (TB), defined as clusters in which the initial case was preceded by at least 24 months of no genotype-matched cases within a geographic area (*1*). This method cannot be applied to endemic clusters (i.e., reported since current TB genotype surveillance began in 2009 with at least 1 case every 24 months) because the initial case cannot be determined. These endemic clusters may be a combination of cases that are the consequence of reactivation of TB in persons who were previously infected and recent transmission of TB.

In this article, we postulate that a statistically driven method can determine the beginning of a TB outbreak in endemic clusters, referred to here as prevalent clusters. Our method searches for instances of excessive unexpected cluster growth above a background rate. We validated our approach by using a combination of epidemiologic data acquired during field investigations and whole-genome sequencing (WGS), which provides higher resolution of the bacterial genome than current genotyping methods (*2*,*3*). Our method systematically reviews data collected at the national level and local epidemiologic data when reported to the Centers for Disease Control and Prevention (CDC).

## The Study

We used the US National Tuberculosis Surveillance System and the National Tuberculosis Genotyping Service datasets for 2009–2016 for this analysis (*4*). We defined prevalent clusters as having >1 TB case with a genotype-matched case also reported in that county during 2009–2010, and subsequent cases reported at least once every 24 months (Technical Appendix). Clusters were reviewed during 2011–2013 for cluster growth. Case counts were aggregated by 3-month time periods, or the first through fourth quarters of each calendar year. We fit negative binomial hurdle models to each consecutive group of eight quarter time intervals and calculated the 95th percentile of the resulting fit (Technical Appendix). Unexpected growth in a prevalent cluster was defined as the earliest quarter where the number of TB cases exceeded the 95th percentile on the basis of fit to the previous 8 quarters (baseline period). For those prevalent clusters identified with unexpected growth, we defined a possible outbreak as a cluster that accrued >10 cases in excess of the quarterly average number of cases in the baseline period during the 3-year follow-up period after unexpected growth was first identified.

When available, we used epidemiologic data from onsite investigations by CDC scientists in conjunction with local TB programs and WGS results to refute or corroborate our classification of possible outbreaks. Studies of epidemiologically linked pairs have estimated *Mycobacterium tuberculosis* to accumulate ≈0.5 single-nucleotide polymorphism (SNP) differences per genome per year (or 1.5 SNPs per 3-year observation period) and found that divergence rarely exceeds 5 SNPs in 3 years between pairs (*5*,*6*). As a conservative estimate in this study, we defined isolates with 2 SNP differences within 3 years to indicate recent transmission (Technical Appendix). We constructed a standard list to meet our definition of an outbreak, consisting of outbreaks investigated by CDC (*7*) and clusters with epidemiologic data and WGS results. Clusters with epidemiologic links or closely related WGS results (i.e., <2 SNP differences) among >50% of cases were corroborated as outbreaks.

Of 2,723 clusters determined during 2011–2016, a total of 706 clusters had >1 TB case in the initial baseline period (2009–2010). Among these 706 prevalent clusters, unexpected growth was identified in 174 (24.6%). Of these clusters, 15 accumulated >10 cases above the baseline average during the 3-year follow-up period after unexpected growth, meeting our definition of a possible outbreak. Of these clusters, 10 had WGS results and epidemiologic data, of which 8 met our definition of an outbreak, 1 was refuted on the basis of diverse WGS results, and 1 was marginal in meeting our definition. The remaining 5 clusters were indeterminate because neither WGS results nor epidemiologic data were available to CDC at the time of publication. When we excluded indeterminate clusters, we found that our methods had a positive predictive value of 80%.

Our standard list included 3 outbreaks that were not detected by our method. Two undetected outbreaks were initially reported during the baseline period (2009–2010) and grew quickly, setting a high starting baseline average. Although our method flagged these clusters for unexpected growth, they did not accumulate sufficient cases during the follow-up period to meet our threshold for excessive growth. Our method identified no unexpected growth in the remaining known outbreak.

**Figure 1:**
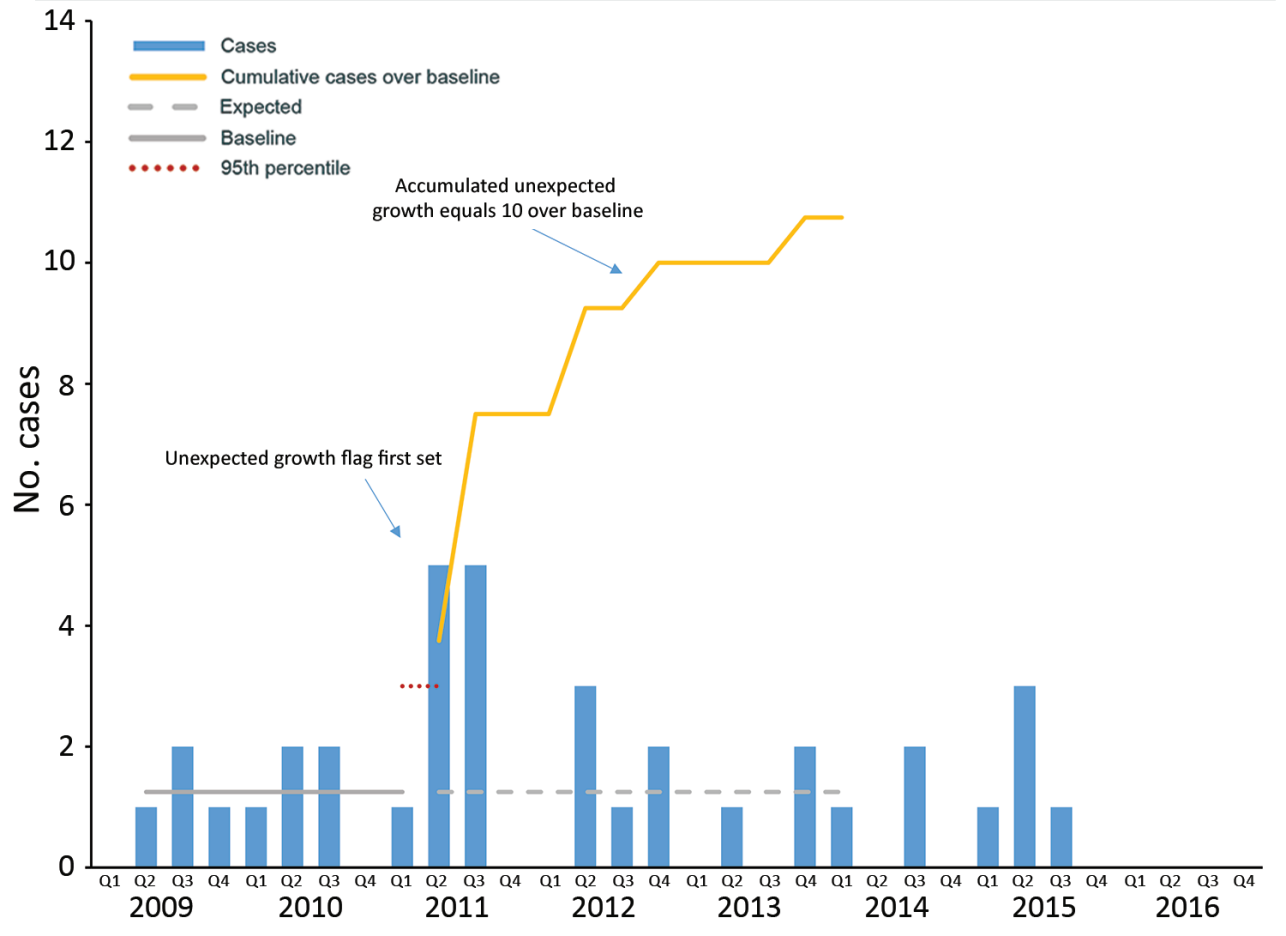
Epidemiologic curve showing a prevalent (endemic) outbreak of tuberculosis, by case counts per 3-month period, United States, 2009–2016. Q, quarter.

We provide an epidemiologic curve (Figure 1) of the marginal cluster detected as a possible outbreak with WGS results and epidemiologic links reported. Our method identified unexpected growth in the second quarter of 2011, with 5 cases exceeding the 95th percentile of the hurdle model for the previous 8 quarters, calculating a baseline average of 1.25 cases per quarter. From this time point, we counted the number of cases that exceeded the baseline average: 3.75 in the second and third quarters of 2011, 1.75 in the second quarter of 2012, 0.75 in the fourth quarter of 2012, and 0.75 in the fourth quarter of 2013. The cluster accumulated 10.75 excess cases within 3 years of unexpected growth and met our criteria as a possible outbreak.

**Figure 2:**
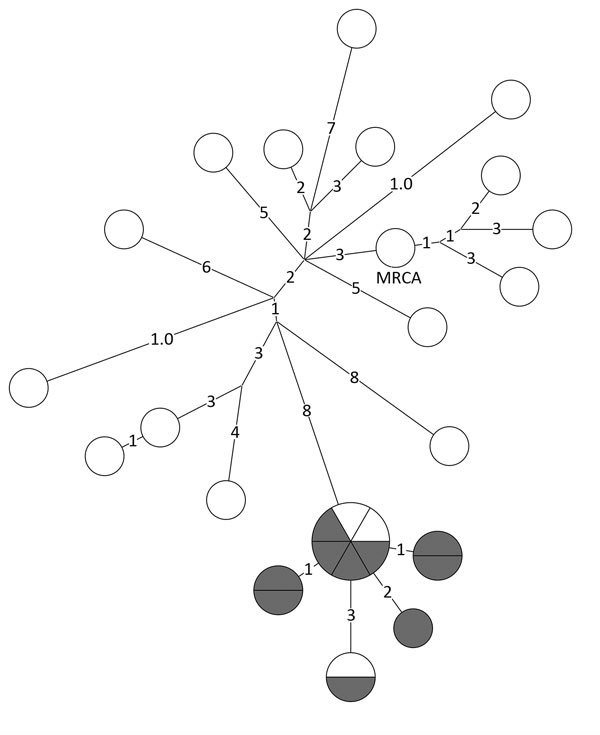
Whole-genome sequencing results for a prevalent (endemic) cluster detected as a possible tuberculosis outbreak, United States, 2009–2016. Values indicate number of SNPs. Shown is a closely related (<2 SNPs) group of 11 isolates (lower section of phylogenetic tree). Isolates reported during a 3-year window of unexpected growth are indicated in gray. One isolate reported 1 quarter before and 1 isolate reported 1 quarter after the 3-year window of unexpected growth detection are indicated in white. An additional 2 isolates were 3 SNPs from this closely related group, 1 during (gray) and 1 outside (white) the unexpected growth window. MRCA, most recent common ancestor; SNP, single-nucleotide polymorphism.

WGS results showed that, of the 20 isolates reported 3 years after unexpected growth, a closely related group of 9 isolates were within 2 SNPs of each other (Figure 2). Two additional isolates within the closely related group were outside the unexpected growth time window, and 2 other isolates, 1 reported during and 1 outside the time window, were within 3 SNPs of the closely related group.

## Conclusions

This research continues our development of alerting clusters of public health concern (*8**–**10*). We describe a statistical method that accurately detected TB outbreaks among endemic clusters. Our method, based on routinely collected surveillance data, can be prospectively implemented to detect possible TB outbreaks. CDC plans to conduct universal WGS for all culture-confirmed TB case specimens, which would provide more precise molecular data for possible outbreaks. Our method will still be helpful in identifying when cluster growth exceeds an expected rate.

Genotype surveillance of TB cases is limited to culture-confirmed cases, which represent 78% of all cases (*11*). Therefore, we excluded non–culture-confirmed cases. In a similar manner, our validation was limited to epidemiologic data available to CDC. In addition, our approach searches for outbreaks within a single county, but TB transmission can cross county borders.

Our method for determining unexpected growth, based on the 95th percentile for a negative binomial hurdle model, serves only as an initial screening. Although our method can identify excessive unexpected growth, to confirm an outbreak requires epidemiologic investigation and increasingly relies on WGS results. Even with universal WGS, outbreak confirmation requires epidemiologic investigations to distinguish recent transmission from reactivation of remotely acquired TB (*12*).

Our methods provide an approach to detect possible outbreaks among prevalent clusters. We expect to incorporate these methods into CDC’s existing surveillance system for large outbreaks of TB in the United States (*13*). We will explore additional approaches to evaluate initial cases of unexpected growth in all clusters, incident and prevalent, to develop an algorithm that can predict which clusters are most likely to become outbreaks.

Technical Appendix Additional information on a statistical method to detect tuberculosis outbreaks among endemic clusters in a low-incidence setting.
