# Supplementary material for: Statistical Method to Detect Tuberculosis Outbreaks among Endemic Clusters in a Low-Incidence Setting
Source: Emerg Infect Dis. 2018 Mar;24(3):573–5. doi: 10.3201/eid2403.171613 (PMC5823347; doi:10.3201/eid2403.171613)
Supplement: Technical Appendix — Additional information on a statistical method to detect tuberculosis outbreaks among endemic clusters in a low-incidence setting. [file 17-1613-Techapp-s1.pdf]

Article DOI: https://doi.org/10.3201/eid2402.171613

Statistical Method to Detect Tuberculosis Outbreaks among Endemic Clusters in a Low-Incidence Setting

Technical Appendix

Additional Methods

Genotyping was performed by combining results of spoligotyping and 24-locus mycobacterial interspersed repetitive unit–variable number tandem repeat analysis (*1*,*2*). A tuberculosis cluster was defined as ≥2 genotype-matched cases reported from the same county or county-equivalent jurisdiction. (The equivalent of a county is a census area in Alaska and a parish in Louisiana.) Incident clusters were defined as those in which the initial case was preceded by a 24-month period of no reported genotype-matched cases from the same county. Prevalent clusters become eligible to be incident clusters if 24 months elapsed with no cases of the same genotype reported in that jurisdiction. A total of 2,017 incident clusters were excluded from our analysis.

All data were imported into and managed in SAS 9.3 (SAS Institute Inc., Cary, NC, USA) for analysis, particularly to designate prevalent from incident clusters and to construct a dataset to identify unexpected growth. Three-month time periods corresponded to each quarter in a calendar year (e.g., cases reported during April–June 2012 corresponded to the second quarter of 2012. Aggregate quarterly cases of each cluster were documented in a spreadsheet and saved as a comma-separated values file (.csv) which included the unique cluster identifier, year and quarter date, and case counts. These data were imported to the open source statistical software R, version 3.3.3 (*3*).

Model fits for case counts were performed by using the packages zoo (*4*) for moving window calculations and pscl (*5*) for hurdle regressions. A negative binomial hurdle model was fit to each successive 8-quarter time-window of case counts. If all 8 quarters had cases (no zero quarters), a straight negative binomial model was fit. The negative binomial distribution closely approximates a Poisson when the dispersion parameter is large. Thus, our fitting procedure was sufficiently flexible to accommodate a negative binomial hurdle, a negative binomial, or a Poisson fit for case counts in each consecutive 8-quarter time-window. We calculated 95th percentiles for each fit. Negative binomial models accommodate over-dispersion (i.e., greater variability than expected on the basis of a given statistical model), which typically occurs in count data. Hurdle models (*6*) account for excess zeros in

count data, which is relevant for tuberculosis, given its generally low levels of incidence, even during outbreaks.

Genomic DNA was extracted from *Mycobacterium tuberculosis* strains by using the Quick-DNA Fungal/Bacterial Kit (Zymo Research Corp., Irvine, CA, USA), and 1 ng was used to prepare sequencing libraries by using the NexteraXT Kit (Illumina, San Diego, CA, USA) according to the package insert. Libraries were sequenced on an Illumina MiSeq instrument to generate 250-bp paired-end reads. The reads were aligned to the reference genome *M. tuberculosis* H37Rv (NC_000962.3) by using Lasergene Genomics Suite (DNASTARar Inc., Madison, WI, USA).

Single-nucleotide polymorphisms (SNPs) were filtered to produce a list of high-quality, informative SNPs for each genotype-matched cluster. SNPs within repeat regions, insertion sequence elements, and the Pro-Pro-Glu (PPE) and Pro-Glu–polymorphic repetitive sequence class (PE-PRGS) gene families were not included. SNPs were then mapped on to a phylogenetic tree by using the neighbor-joining method in BioNumerics 7.6.2 (Applied Maths, Sint-Martens-Latem, Belgium). We designated isolates as being closely related if they were within 2 SNP differences of each other within 3 years of being reported. Although there is no consensus in the literature regarding how many SNP differences would be considered as standard for likelihood of recent transmission, our designation of 2 SNPs within 3 years is a conservative estimate (*7*–*9*).

References

1. Supply P, Allix C, Lesjean S, Cardoso-Oelemann M, Rüsch-Gerdes S, Willery E, et al. Proposal for standardization of optimized mycobacterial interspersed repetitive unit-variable-number tandem repeat typing of *Mycobacterium tuberculosis.* J Clin Microbiol. 2006;44:4498–510. PubMed http://dx.doi.org/10.1128/JCM.01392-06

2. Allix-Béguec C, Fauville-Dufaux M, Supply P. Three-year population-based evaluation of standardized mycobacterial interspersed repetitive-unit-variable-number tandem-repeat typing of *Mycobacterium tuberculosis.* J Clin Microbiol. 2008;46:1398–406. PubMed http://dx.doi.org/10.1128/JCM.02089-07

3. R Core Team. R: a language and environment for statistical computing. Vienna: R Foundation for Statistical Computing, 2017 [cited 2017 Sep 12] http://www.R-project.org/

4. Zeileis A, Grothendieck G. Zoo: S3 infrastructure for regular and irregular time series. J Stat Softw. 2005;14:1–27. http://dx.doi.org/10.18637/jss.v014.i06

5. Zeileis A, Kleiber C, Jackman S. Regression models for count data in R. Journal of Statistical Software. 2008;27 [cited 2017 Dec 15] http://www.jstatsoft.org/v27/i08/

6. Mullahy J. Specification and testing of some modified count data models. J Econometrics. 1986;33:341–65. http://dx.doi.org/10.1016/0304-4076(86)90002-3

7. Gardy JL, Johnston JC, Ho Sui SJ, Cook VJ, Shah L, Brodkin E, et al. Whole-genome sequencing and social-network analysis of a tuberculosis outbreak. N Engl J Med. 2011;364:730–9. PubMed http://dx.doi.org/10.1056/NEJMoa1003176

8. Kato-Maeda M, Ho C, Passarelli B, Banaei N, Grinsdale J, Flores L, et al. Use of whole genome sequencing to determine the microevolution of *Mycobacterium tuberculosis* during an outbreak. PLoS One. 2013;8:e58235. PubMed http://dx.doi.org/10.1371/journal.pone.0058235

9. Walker TM, Monk P, Smith EG, Peto TE. Contact investigations for outbreaks of *Mycobacterium tuberculosis*: advances through whole genome sequencing. Clin Microbiol Infect. 2013;19:796–802. PubMed http://dx.doi.org/10.1111/1469-0691.12183
